# Detection of acute myocardial infarct with T1-mapping post ferumoxytol contrast administration

**DOI:** 10.1186/1532-429X-15-S1-P173

**Published:** 2013-01-30

**Authors:** Deneen Spatz, Igor Klem, Lowie M Van Assche, Enn-Ling Chen, Wolfgang G Rehwald, Han W Kim, Christoph J Jensen, David Wendell, Elizabeth Jenista, Raymond J Kim

**Affiliations:** 1 Duke University Cardiac MRI, Durham, NC, USA; 2 Seimens, Chicago, IL, USA

## Background

Gadolinium-enhanced CMR can be a useful adjunct in the diagnosis and assessment of acute myocardial infarction (AMI). However, gadolinium contrast is contraindicated in severe renal impairment. Alternative, non-gadolinium contrast agents for AMI assessment and contrast-enhanced CMR in general would be of substantial clinical importance. Ferumoxytol, an intravenous iron-supplementation drug, was approved in 2009 by the FDA for treatment of iron-deficiency anemia in patients with chronic kidney disease, and is known to have T1 shortening properties. The utility of T1-weighted ferumoxytol-enhanced CMR for detecting AMI has not been previously evaluated.

## Methods

Six canines underwent acute LAD occlusion (70-minutes) with reperfusion. In-vivo CMR was performed four days post-MI on a 3.0T scanner in five animals (one animal died prior to acquisition of images). Clinically available Ferumoxytol (30mg Fe/ml) was administered to provide 5 mg Fe/kg body weight. Short-axis T1-maps were acquired using a single-shot inversion-recovery, gradient-echo sequence with >20 inversion times ranging from 100-3000 ms. Acquisitions were separated by >10-second delay to allow full recovery of magnetization, and T1 times were determined by standard 3-parameter iterative curve-fitting. T1 maps were acquired serially at multiple time-points (5-8) following Ferumoxytol-injection (range 1-24 hours). Following imaging the hearts were removed for histopathologic analysis and the presence, location, and extent of AMI was validated by 2, 3, 5-triphenyl-tetrazolium chloride (TTC) staining.

## Results

**Figure 1:**
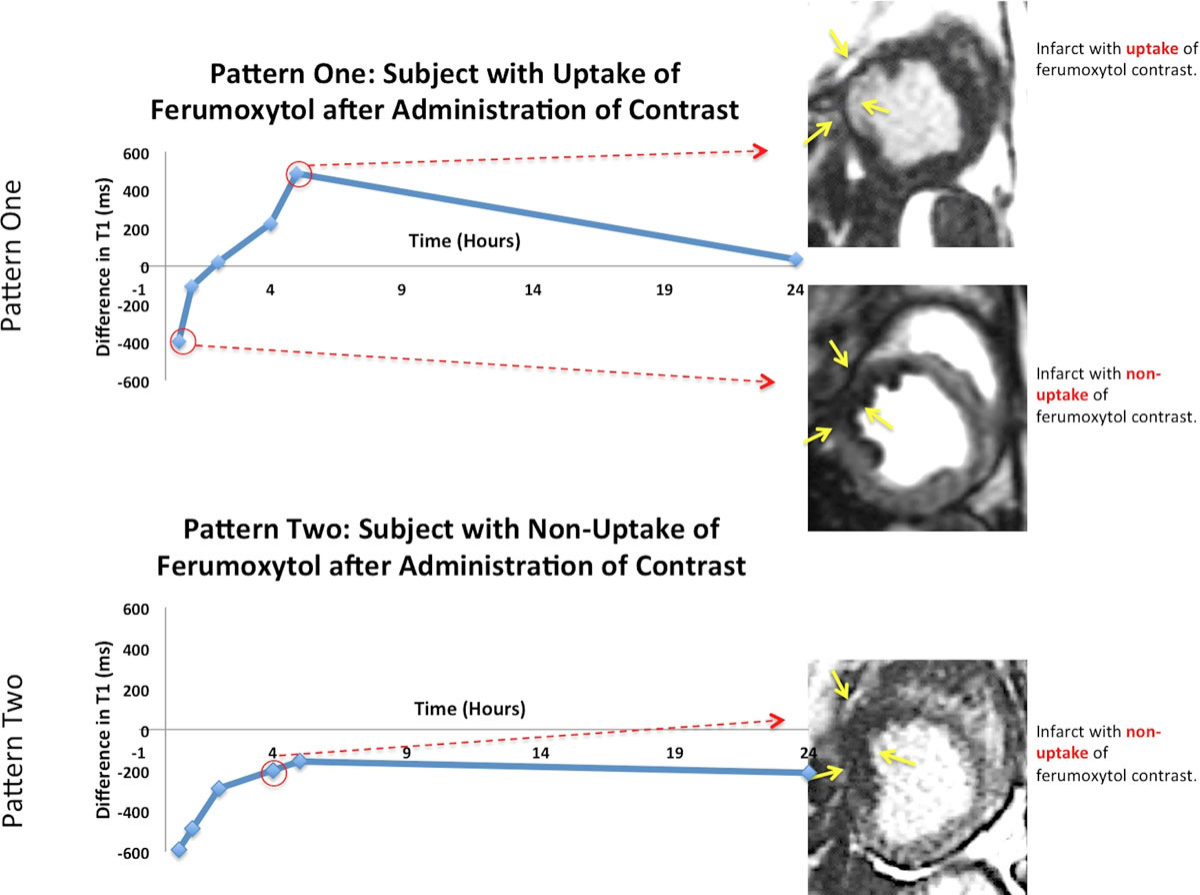
Difference between normal minus infarcted myocardium. Pattern one shows the uptake of ferumoxytol contrast. At time 30 min there is non-uptake of contrast - infarct appears black or hypo-intense. At time 5 hours there is uptake of contrast - infarct appears whit or hyper-intense. At 24 hours the contrast has washed out of the infarct.

**Figure 2:**
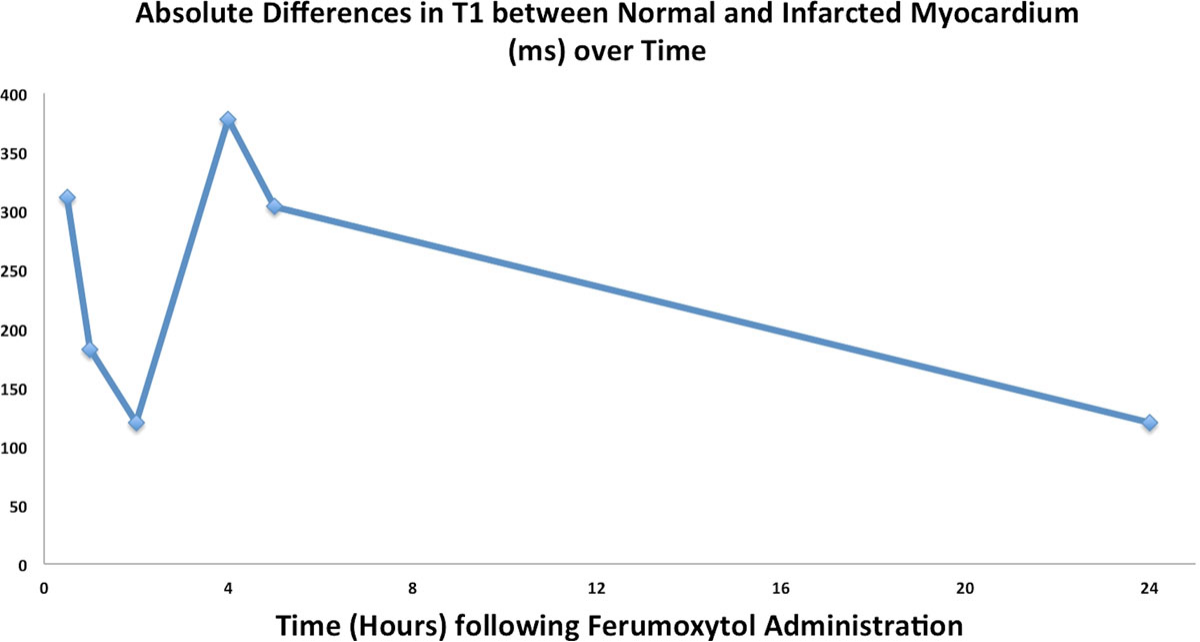
Absolute Difference in T1 between Normal and Infarcted Myocardium (ms) over Time after administration of Ferumoxytol contrast.

All five animals had AMI verified by pathology, and mean infarct size was 12.7% (range 2.6-16.8%) of LV mass. Ferumoxytol-kinetics of AMI was complex following two patterns (Figure 1, top). Pattern one (n=3) demonstrated an early restriction of Ferumoxytol uptake in AMI regions (approx. 0-2 hrs post Ferumoxytol) compared with normal myocardium followed later by increased uptake in AMI relative to normal (approx. 2-6 hrs post), and finally washout of Ferumoxytol in both AMI and normal myocardium (24 hrs post). Pattern two (n=2) demonstrated reduced Ferumoxytol uptake compared with normal myocardium throughout all time points (Figure 1, bottom). The absolute difference in T1 between normal myocardium and AMI over time averaged for the five animals is shown in Figure 2. In 20 of 27 time-points (74%), the absolute T1-difference was >100ms, which was sufficient to visualize AMI on routine inversion-recovery CMR.

## Conclusions

Ferumoxytol kinetics in AMI are complex and may reflect either reduced or increased contrast uptake depending on time post contrast administration. However, AMI can consistently be detected by Ferumoxytol-enhanced CMR given positive absolute differences in T1.

## Funding

5ROIHL064726-07

